# Associations of psychological factors with atherosclerosis and cardiovascular health in middle-age: the population-based Swedish CArdioPulmonary bioImage study (SCAPIS)

**DOI:** 10.1186/s12889-024-18924-w

**Published:** 2024-05-30

**Authors:** Sara Higueras-Fresnillo, Ángel Herraiz-Adillo, Viktor H. Ahlqvist, Robin Öberg, Cecilia Lenander, Patrik Wennberg, Josefin Wångdahl, Daniel Berglind, Bledar Daka, Carl Johan Östgren, Karin Rådholm, Pontus Henriksson

**Affiliations:** 1 Department of Health, Medicine and Caring Sciences, Linköping University, Linköping, Sweden; 2 Department of Preventive Medicine and Public Health, Universidad Autónoma de Madrid, Madrid, Spain; 3 Department of Biomedicine, Aarhus University, Aarhus, Denmark; 4 Department of Clinical Sciences in Malmö, Centre for Primary Health Care Research, Lund University, Lund, Sweden; 5 Department of Public Health and Clinical Medicine, Umeå University, Umeå, Sweden; 6 Aging Research Center, Karolinska Institutet and Stockholm University, Stockholm, Sweden; 7 Department of Public Health and Caring Sciences, Uppsala University, Uppsala, Sweden; 8 Centre for Epidemiology and Community Medicine, Region Stockholm, Stockholm, Sweden; 9 Family medicine, School of Public Health and Community Medicine, Institute of Medicine, Sahlgrenska Academy, University of Gothenburg, Gothenburg, Sweden; 10 Centre of Medical Image Science and Visualization (CMIV), Linköping University, Linköping, Sweden; 11 The George Institute for Global Health, University of New South Wales, Sydney, Australia; 12 Institute of Environmental Medicine, Karolinska Institutet, Stockholm, Sweden

**Keywords:** Atherosclerosis, Cardiovascular disease, Coronary artery calcification, Coronary computed tomography angiography, Life’s Essential 8, Middle-aged, Psychological factors, SCAPIS

## Abstract

**Background:**

Cardiovascular disease (CVD) is a major global health issue, primarily caused by atherosclerosis. Psychological factors may play a role in the development and progression of CVD. However, the relationship between psychological factors and atherosclerosis is complex and poorly understood. This study, therefore, aimed to examine the association of psychological factors with (i) coronary and carotid atherosclerosis and (ii) cardiovascular health according to Life’s Essential 8, in a large Swedish cohort.

**Methods:**

This study utilized data from the Swedish CArdioPulmonary bioImage Study (SCAPIS), a large population-based project including individuals aged 50 to 65 years. Several psychological factors were analysed: general stress, stress at work, financial stress, major adverse life events, locus of control, feeling depressed, and depression. Coronary atherosclerosis was assessed as the degree of stenosis by coronary computed tomography angiography (CCTA) and coronary artery calcification (CAC) scores. Carotid atherosclerosis was examined using ultrasound. In addition, cardiovascular health was examined using the Life’s Essential 8 concept created by the American Heart Association, which includes four health behaviors and four health factors. Associations were examined through binomial logistic regression (atherosclerosis variables) and linear regression (Life’s Essential 8).

**Results:**

A total of 25,658 participants were included in the study. The presence of financial stress, higher locus of control, and depression was weakly associated with increased odds of CCTA stenosis, CAC ≥ 1 and the presence of carotid plaques (all odds ratios: 1.10–1.21, 95% CI: 1.02–1.32) after adjusting for sex, age, and study site. However, these associations were attenuated and not statistically significant after additional adjustments for socioeconomic factors and health behaviors. Conversely, we observed inverse associations between the worst category for all psychological factors and cardiovascular health according to Life’s Essential 8 score (all standardized β-Coefficient ≤-0.033, *p* < 0.001).

**Conclusion:**

While there were no strong and consistent associations between psychological factors and atherosclerosis, the consistent associations of psychological factors with cardiovascular health by Life’s Essential 8 may have relevance for future CVD risk. However, further studies are needed to elucidate the long-term effects of psychological factors on atherosclerosis development and cardiovascular health.

**Supplementary Information:**

The online version contains supplementary material available at 10.1186/s12889-024-18924-w.

## Background

Cardiovascular disease (CVD) is a leading cause of morbidity and mortality worldwide, and the prevalence of CVD continues to increase, despite advances in medical treatments and interventions [1, 2]. An underlying pathological basis of CVD is atherosclerosis, a chronic inflammatory disease characterized by the build-up of plaques in arterial walls, leading to the narrowing and hardening of the arteries [3]. While traditional risk factors for atherosclerosis, including smoking, hypertension, dyslipidaemia, or diabetes, are well established [4–8], recent research has highlighted the potential role of psychological factors in the development and progression of CVD, emphasizing the concept of integral health [9, 10]. However, the relationship between psychological factors and atherosclerosis is complex and multifaceted, and several mechanisms have been proposed to explain this association. For instance, psychological stress appears to cause increased sympathetic activity, which, in turn, may lead to an increase in cardiovascular risk factors and biological dysfunction [11–13]. Furthermore, a potential mechanism linking psychological factors and atherosclerosis is through the influence of psychological stress on health behaviors, such as diet, physical activity, and sleep [14]. For example, individuals experiencing high levels of stress may engage in unhealthy behaviors that contribute to the development of atherosclerosis.

Although several studies have suggested that chronic stress, depression, and anxiety may play a role in the development and progression of subclinical atherosclerosis, the evidence is not consistent [15, 16]. A few studies have investigated the association between psychological factors and subclinical atherosclerosis measured as coronary artery calcification (CAC), carotid intima media thickness (CIMT) and ankle-brachial index [16–19]. However, these studies did not use the non-invasive gold standard coronary computed tomography angiography (CCTA) imaging technique. In contrast to CAC, which only provides information about calcified plaques, CCTA provides anatomical information of both calcified and non-calcified plaques and stenosis [20]. This increased sensitivity for detecting CVD makes CCTA a more reliable imaging tool for assessing the presence and severity of subclinical atherosclerosis in patients with psychological risk factors [21].

Therefore, the aim of this study, conducted within the large population-based Swedish CArdioPulmonary bioImage Study (SCAPIS) was twofold: (i) to examine the association between psychological factors and coronary and carotid atherosclerosis and (ii) to examine the association between psychological factors and cardiovascular health according to Life’s Essential 8 (LE8).

## Methods

### Study design and population

We used data from SCAPIS, including 30,154 individuals (51.4% women) aged 50 to 64 years randomly recruited from 6 university cities in Sweden (Gothenburg, Linköping, Malmö/Lund, Stockholm, Umeå, and Uppsala) [3, 22]. Study methods have been reported in detail elsewhere [22]. The SCAPIS project aimed to characterize a large general population sample to improve prevention strategies for CVD. Participants were included between 2013 and 2018; the final study response rate was 50.3%.

Of the 30,154 participants in the SCAPIS project, 25,658 participants had data on at least one psychological factor and had complete covariates. Additionally, 3,610 participants had missing data on any of the four proximal coronary segments, leaving a final sample size of 22,048, 21,727, 25,534, and 25,622 participants with data on CCTA coronary stenosis, CAC score, carotid plaque and LE8 score, respectively (see Supplementary Fig. 1).

#### Ethical approval

was obtained from the Swedish Ethical Review Board (reference numbers: 2021-06408-01 and 2022-04375-02), and all participants provided written informed consent.

### Psychological factors

The present study analysed several psychological variables, related to stress and mental health, that have been previously associated to cardiovascular diseases [15] including: general stress, stress at work, financial stress, major adverse life events, locus of control, feeling depressed, and depression. General stress was defined as “feeling tense, irritable, anxious, or having sleep problems due to conditions at work or home” [15]. Stress at work was assessed with an adaptation of the Copenhagen Psychosocial Questionnaire [23]. The original version comprises 30 questions. In SCAPIS, 10 questions were selected, and the following three main clusters of scales were developed: (i) demands at work, (ii) work organization and content, and (iii) interpersonal relations and leadership. Participants were asked to report how frequently they had felt stressed for each question, using the following response options: a) always; b) often; c) sometimes; d) seldom; e) and never. The score was added to a score of 0–40, and responses were classified as 0–10 never; 11–20 sometimes; ≥ 21 several or permanent. We analysed the level of financial stress through two questions: a) ‘If you should suddenly find yourself in a situation where you had to find 20,000 Swedish kronor in one week, would you manage it?’ (yes was scored as 0 and no as 1) and b) ‘During the last 12 months, have you ever had difficulty managing regular expenses for food, rent, bills, etcetera?’ (yes was scored as 1 and no as 0). The scores were then summed up to create a financial stress score categorized as 0 for ‘no/low,’ 1 for ‘medium,’ and 2 for ‘high’ financial stress [15]. The occurrence of major adverse life events was assessed by asking participants if they had experienced any of the following life events in the previous 12 months or before: serious illness or accident in the family, concerns for someone close, death of someone close, own divorce or separation, had to change housing, had to change job, loss of job, felt insecure at work, serious financial problems, or received a criminal penalty. In the main analysis, only major adverse life events occurred in the last 12 months were considered. We used a questionnaire to determine the generalized locus of control or the perception of being able to control life circumstances in six areas (at work, in life, positive expectations in the next 5–10 years, feeling of being treated unfairly, unexpected changes in the last 10 years, and giving up trying to improve in life for a long time). To create a summary score, Likert-type responses of the 6 components in the locus of control were scored from 1 to 6, resulting in a total score ranging from 1 to 36, which was divided into quartiles. The first quartile represented the highest score (more internal control) and the fourth quartile the lowest locus of control (less internal control). Feeling depressed was assessed by asking if the participant had felt sad, blue, or depressed for two weeks or more in a row in the previous 12 months and if the answer was yes, a set of 7 additional questions were asked: loss of interest in things, feeling tired or having low energy, weight gain or loss, difficulty falling asleep, difficulty concentrating, thinking about death, and feeling worthless. For this set of questions, five or more affirmative responses were categorised as depression [15].

### Subclinical atherosclerosis

The SCAPIS imaging protocol has been described in detail elsewhere and meets relevant guidelines [22]. In accordance with the Society of Cardiovascular Computed Tomography guidelines, an 18-segment coronary artery tree model was used to report coronary atherosclerosis from CCTA [24]. Only the 11 most relevant segments were considered in the analysis. Each segment was classified into categories including no stenosis, 1–49% stenosis, ≥ 50% stenosis, not assessable due to calcium blooming, technical failure, or segment missing [3]. Calcium blooming was rated as “1–49% stenosis” due to its tendency to overestimate stenosis, and the presence of a stent was rated as “≥ 50% stenosis”. In the present study, the participant’s level of coronary stenosis was dichotomized as no stenosis or ≥ 1% stenosis based on the segment with the highest level of stenosis. CAC images were analysed using an international standard protocol, and the calcium content in each coronary artery was measured to produce a total CAC score in Agatston units. CAC scores were categorized as 0 or ≥ 1 Agatston units [25, 26].

For carotid artery imaging, a standardized protocol was used with a Siemens Acuson S2000 ultrasound scanner equipped with a 9L4 linear transducer (Siemens, Forchheim, Germany) and analysed by regularly trained operators [22, 27]. Two-dimensional greyscale images were used to identify extracranial carotid plaques in the common carotid artery, bulb, and internal carotid artery on both the right and left sides. Only participants with valid readings for both carotid arteries were included in the analysis. In accordance with the Mannheim consensus, carotid plaque was defined as “any focal structure that encroaches into the arterial lumen of at least 0.5 mm or 50% of the surrounding intima-media thickness value or demonstrates a thickness > 1.5 mm as measured from the media-adventitia interface to the intima-lumen interface” [27]. Participants in SCAPIS were classified as having no plaque or any carotid plaque; based on the absence or presence of carotid plaques.

### Covariates

Age, sex (woman or man), site (Gothenburg, Linköping, Malmö/Lund, Stockholm, Umeå, and Uppsala), educational level (achieved highest level of education: no formal, primary, secondary, or university level), current marital status (single, divorced, married, or widow/widower) and alcohol intake (frequency, and number of drinks in a typical day) were registered.

### Cardiovascular health assessed by Life’s Essential 8

Associations with health behaviors and health factors which are linked to CVD were assessed using the LE8 cardiovascular health score developed by the American Heart Association [28]. The LE8 score consists of four behaviors (diet, physical activity, nicotine exposure, and sleep health), and four factors (body mass index [BMI], non-high-density lipoprotein cholesterol [non-HDL], blood glucose, and blood pressure).

A detailed protocol about the measurement and calculation of LE8 health behaviors and health factors in SCAPIS has been previously published [29]. Diet score was calculated using the Mediterranean Eating Pattern for Americans score [30] using a web-based questionnaire (MiniMeal-Q), and physical activity was assessed using a tri-axial accelerometer (Actigraph GT3X+, wGT3X+, and wGT3X-BT) worn by participants during seven days [31]. Nicotine exposure was assessed using questions about the current and former smoking status, and information about the smoking habit of cohabitants. Sleep health was measured by asking about the number of hours of sleep per night and problems during sleep time.

Factors were measured using standardized methods and laboratory techniques for BMI, blood lipids, blood glucose, and blood pressure [29]. According to the American Heart Association, the 8 components of LE8 were ranked from 0 to 100, with the higher scores indicating better cardiovascular health. In consonance with the American Heart Association, the LE8 score was calculated as the unweighted average of all present components, and a minimum of 7 components was considered necessary to compute the overall LE8 score.

Furthermore, two separate scores were calculated for LE8 behaviors and LE8 factors, with a range from 0 to 100. These scores were determined by taking the unweighted average of all present components in behaviors and factors, respectively.

### Statistical analysis

We conducted a complete case analysis on all 25,658 participants having data on at least one psychological factor, and complete covariates. Descriptive characteristics of study participants are presented as mean ± standard deviation or frequencies (percentages). The associations between psychological factors and coronary atherosclerosis and carotid plaques were analysed through logistic regression and expressed as odds ratios (ORs) with their 95% confidence intervals (95% CI). For each psychological factor we fit an individual model. Three models with progressive adjustment for potential confounders were fitted. Model 1 was adjusted for sex, age, and site and model 2 was adjusted for model 1 plus educational level and marital status. To examine whether the association between psychological factors and subclinical atherosclerosis was independent of health behaviors, we also fitted a model 3 in which we accounted for model 2 plus alcohol, smoking, BMI, diet, physical activity, and sleep. A linear regression analysis was done as well, adjusted for model 2 (sex, age, site, educational level, and marital status), to examine the associations of psychological factors on cardiovascular health according to LE8 components. The results are presented in the form of standardized beta coefficients (β) and their corresponding p-values. To test whether sex or socioeconomic status modify the association between psychological factors and the outcomes (atherosclerosis y LE8 score), we examined the interactions between psychological factors in relation to sex, education level and unemployment in adjusted models (model 2 and model 3). In general, we found very little evidence of an interaction between phycological factors and sex, educational level, or unemployment in relation to atherosclerosis or cardiovascular health. Therefore, we performed the analyses without stratifying the sample. Finally, we performed a sensitivity analysis for the associations of a stressful life event on life events that occurred less or more than 12 months ago. The analyses were performed using IBM SPSS version 28 (Armonk, NY: IBM Corp). All statistical tests were two-sided and *p* < 0.05 was considered statistically significant.

## Results

### Descriptive statistics

Table 1 presents descriptive data on the study population. A total of 25,658 participants were included in the analyses, with 13,388 women (52.2%) and 12,270 men (47.8%). The mean age of the population was 57.5 ± 4.3 years. Approximately, 12.0% of the participants were current smokers, while 36.6% were ex-smokers, and more than half had never smoked. Almost half of the participants had a university degree, and 74.8% of the participants were married. In relation to psychological factors, most participants reported experiencing periods of stress in their lives, both overall and at work, with a high percentage reporting permanent stress at work. Feeling depressed was reported by 27.7% of the participants, with a higher proportion among women compared to men (34.6% vs. 20.1%). Among those who presented depressive feelings, 14.9% of the participants reported having 5 or more indicators of depression.

**Table 1:** Clinical characteristics of the study sample by sex

	Total *n* = 25,658	Women *n* = 13,388	Men *n* = 12,270
**Age and cardiovascular risk factors**			
Age, y	57.5 ± 4.3	57.5 ± 4.3	57.5 ± 4.4
BMI, kg/m^2^	26.9 ± 4.4	26.5 ± 4.8	27.4 ± 3.9
Total cholesterol, mg/dL	212.5 ± 40.4	218.3 ± 39.3	206.1 ± 40.7
Systolic blood pressure, mmHg	125.8 ± 17.0	123.1 ± 17.8	128.8 ± 15.6
Diastolic blood pressure, mmHg	77.5 ± 10.5	76.6 ± 10.8	78.4 ± 10.2
Fasting glucose, mg/dL	103.1 ± 19.5	99.9 ± 17.0	106.6 ± 21.4
HbA1c, mmol/mol	36.4 ± 6.2	36.2 ± 5.5	36.7 ± 6.9
MVPA, min/day	56.0 ± 29.5	54.1 ± 27.7	58.1 ± 31.2
Diet, MEPA score (0-16)	8.2 ± 2.1	8.8 ± 2.1	7.7 ± 2.0
**Smoking, n (%)**	*n* = 25,658	*n* = 13,388	*n* = 12,270
Current	3083 (12.0)	1644 (12.3)	1439 (11.7)
Ex-smoker	9383 (36.6)	5235 (39.1)	4148 (33.8)
Never	13,192 (51.4)	6509 (48.6)	6683 (54.5)
**Alcohol intake, last year, n (%)**	*n* = 25,564	*n* = 13,339	*n* = 12,225
Never	2130 (8.3)	1293 (9.7)	837 (6.8)
Monthly or less	3874 (15.2)	2360 (17.7)	1514 (12.4)
2–4 times a month	9809 (38.4)	5127 (38.4)	4682 (38.3)
2–3 times a week	7904 (30.9)	3889 (29.2)	4015 (32.8)
≥4 times a week	1847 (7.2)	670 (5.0)	1177 (9.6)
**Education level**	*n* = 25,658	*n* = 13,388	*n* = 12,270
Less than primary school	132 (0.5)	72 (0.5)	60 (0.5)
Primary school	2072 (8.1)	955 (7.1)	1117 (9.1)
Secondary school	11,605 (45.2)	5692 (42.5)	5913 (48.2)
University degree	11,849 (46.2)	6669 (49.8)	5180 (42.2)
**Current marital status**	*n* = 25,658	*n* = 13,388	*n* = 12,270
Single	3253 (12.7)	1790 (13.4)	1463 (11.9)
Divorced	2803 (10.9)	1837 (13.7)	966 (7.9)
Married	19,181 (74.8)	9444 (70.5)	9737 (79.4)
Widow (-er)	421 (1.6)	317 (2.4)	104 (0.8)
**Psychological factors**			
**General stress**	*n* = 25,479	*n* = 13,285	*n* = 12,194
Never or one time	10,169 (39.9)	4707 (35.4)	5462 (44.8)
Some stressful periods	9927 (39.0)	5165 (38.9)	4762 (39.1)
Constant stress	5383 (21.1)	3413 (25.7)	1970 (16.2)
**Stress at work**	*n* = 21,588	*n* = 11,119	*n* = 10,469
Never	2664 (12.3)	1117 (10.0)	1547 (14.8)
Sometimes	14,363 (66.5)	7047 (63.4)	7316 (69.9)
Several or permanent	4561 (21.1)	2955 (26.6)	1606 (15.3)
**Financial stress**	*n* = 25,144	*n* = 13,099	*n* = 12,045
Little or none	22,893 (91.0)	11,770 (89.9)	11,123 (92.3)
Moderate or severe	2251 (9.0)	1329 (10.1)	922 (7.7)
**Stressful life events**	*n* = 25,359	*n* = 13,206	*n* = 12,153
None	10,093 (39.8)	4763 (36.1)	5330 (43.9)
1	6956 (27.4)	3669 (27.8)	3287 (27.0)
2 or more	8310 (32.8)	4774 (36.2)	3536 (29.1)
**Locus of control**	*n* = 25,123	*n* = 13,080	*n* = 12,043
Q1 (more internal)	8663 (34.5)	4673 (35.7)	3990 (33.1)
Q2	6510 (25.9)	3292 (25.2)	3218 (26.7)
Q3	6349 (25.3)	3196 (24.4)	3153 (26.2)
Q4 (less internal)	3601 (14.3)	1919 (14.7)	1682 (14.0)
**Feeling depressed**	*n* = 25,362	*n* = 13,205	*n* = 12,157
No	18,347 (72.3)	8631 (65.4)	9716 (79.9)
Yes	7015 (27.7)	4574 (34.6)	2441 (20.1)
**Depression**	*n* = 25,310	*n* = 13,178	*n* = 12,132
Not depressed	18,347 (72.5)	8631 (65.5)	9716 (80.1)
0–4	3195 (12.6)	1915 (14.5)	1280 (10.6)
5 o more	3768 (14.9)	2632 (20.0)	1136 (9.4)
**Subclinical atherosclerosis**			
**CCTA stenosis**	*n* = 22,048	*n* = 11,229	*n* = 10,819
No stenosis	12,775 (57.9)	7963 (70.9)	4812 (44.5)
Any stenosis ≥ 1%	9273 (42.1)	3266 (29.1)	6007 (55.5)
**CAC score**	*n* = 21,727	*n* = 11,146	*n* = 10,581
0	13,070 (60.2)	8123 (72.9)	4947 (46.8)
≥ 1 Agatston units	8657 (39.8)	3023 (27.1)	5634 (53.2)
**Carotid plaques**	*n* = 25,534	*n* = 13,321	*n* = 12,213
No plaque	11,509 (45.1)	6777 (50.9)	4732 (38.7)
Any plaque	14,025 (54.9)	6544 (49.1)	7481 (61.3)
**Life’s Essential 8**			
Total score	70.9 ± 11.4	72.8 ± 11.5	68.7 ± 10.8
Behavior score	74.8 ± 11.6	75.6 ± 11.7	74.0 ± 11.4
Factor score	66.9 ± 18.0	70.1 ± 18.2	63.5 ± 17.1

Data refer to mean ± standard deviation or frequencies (percentage). BMI: body mass index, HbA1c: glycated haemoglobin; MVPA: moderate-vigorous physical activity; MEPA: Mediterranean Eating Pattern for Americans; CCTA: coronary computed tomographic angiography, CAC: coronary artery calcium

For subclinical atherosclerosis, 42.1% of participants had any coronary stenosis measured by CCTA, with a higher proportion in men (55.5%) than women (29.1%). Similarly, CAC score ≥ 1 was found in 39.8% of participants, with a higher proportion of men (53.2%) than women (27.1%). In addition, some type of carotid plaques was present in 54.9% of the participants.

### Psychological factors and coronary atherosclerosis

**Fig. 1:**
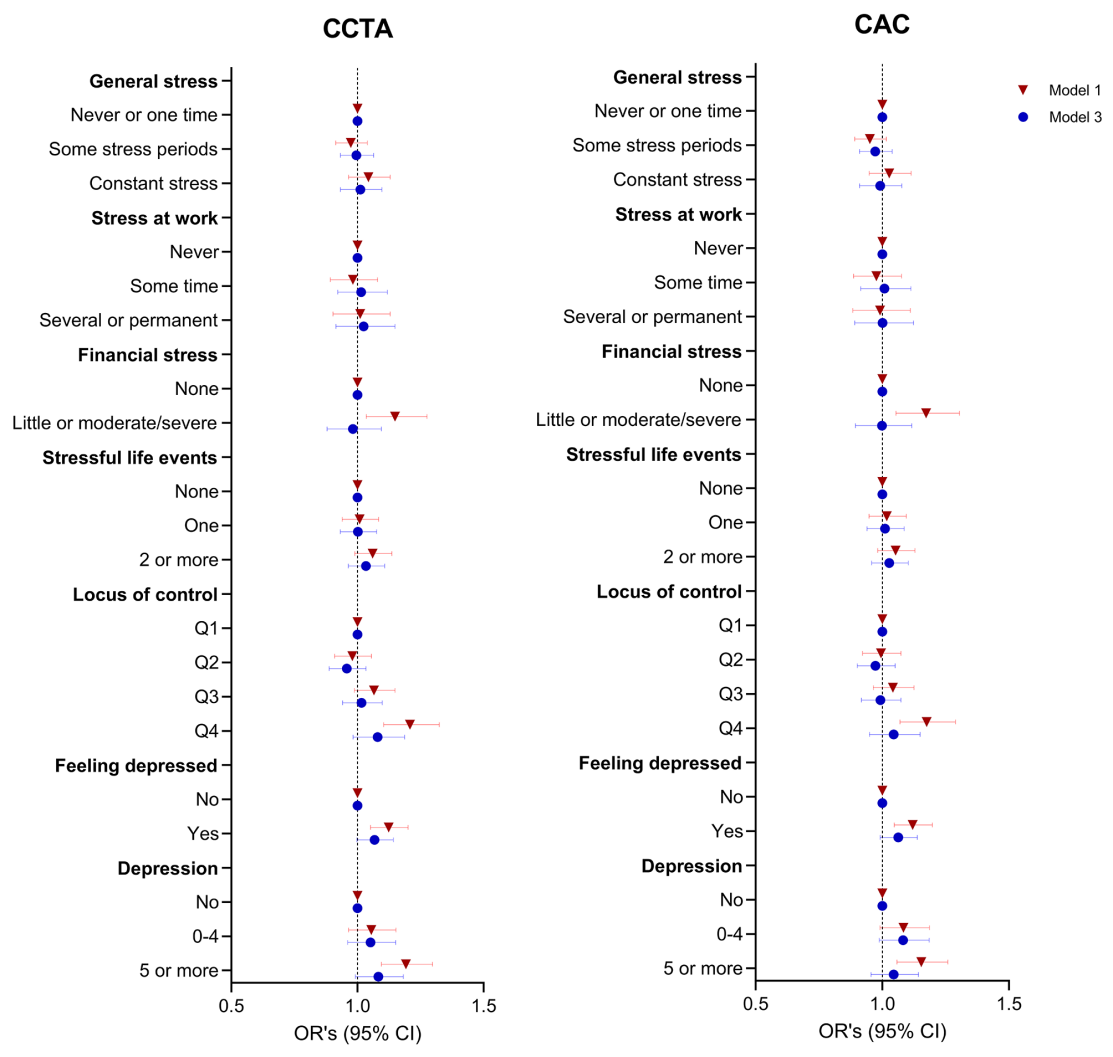
Psychological factors and coronary atherosclerosis The logistic regression models show the odds ratios for the associations between psychological factors and (coronary computed tomographic angiography) CCTA stenosis and (coronary artery calcium) CAC scores ≥ 1. Model 1 adjusted for sex, age, and site; Model 3 adjusted for model 1 plus educational level, marital status, alcohol, smoking, BMI, diet, physical activity, and sleep

Figure 1 presents data on the associations between psychological factors and subclinical coronary atherosclerosis measured as CCTA stenosis and CAC scores (detailed data in Supplementary Table 1). In model 1, general stress or stress at work did not show a statistically significant association with subclinical coronary atherosclerosis. However, individuals experiencing little or moderate/severe financial stress had significantly greater odds of having CCTA stenosis (OR: 1.15, 95% CI: 1.04–1.28) and an elevated CAC score (OR: 1.17, 95% CI: 1.05–1.31). Furthermore, the highest category of locus of control (more internal) was associated with greater odds of CCTA stenosis (OR: 1.21, 95% CI: 1.10–1.32) and an elevated CAC score (OR: 1.18, 95% CI: 1.07–1.29) compared to the lowest category. Finally, feeling depressed was related to have higher ORs of CCTA stenosis (OR: 1.12, 95% CI: 1.05–1.20) and an elevated CAC score (OR: 1.12, 95% CI: 1.05–1.20). However, all the abovementioned associations in model 1 for CCTA stenosis and CAC were attenuated and not statistically significant in model 3. In a sensitivity analysis, we separated the associations of a stressful life event into life events that took place less or more than 12 months ago (Supplementary Table 2) and in agreement with our main analyses, there were no associations between stressful life events and CCTA stenosis.

### Psychological factors and carotid atherosclerosis

**Fig. 2:**
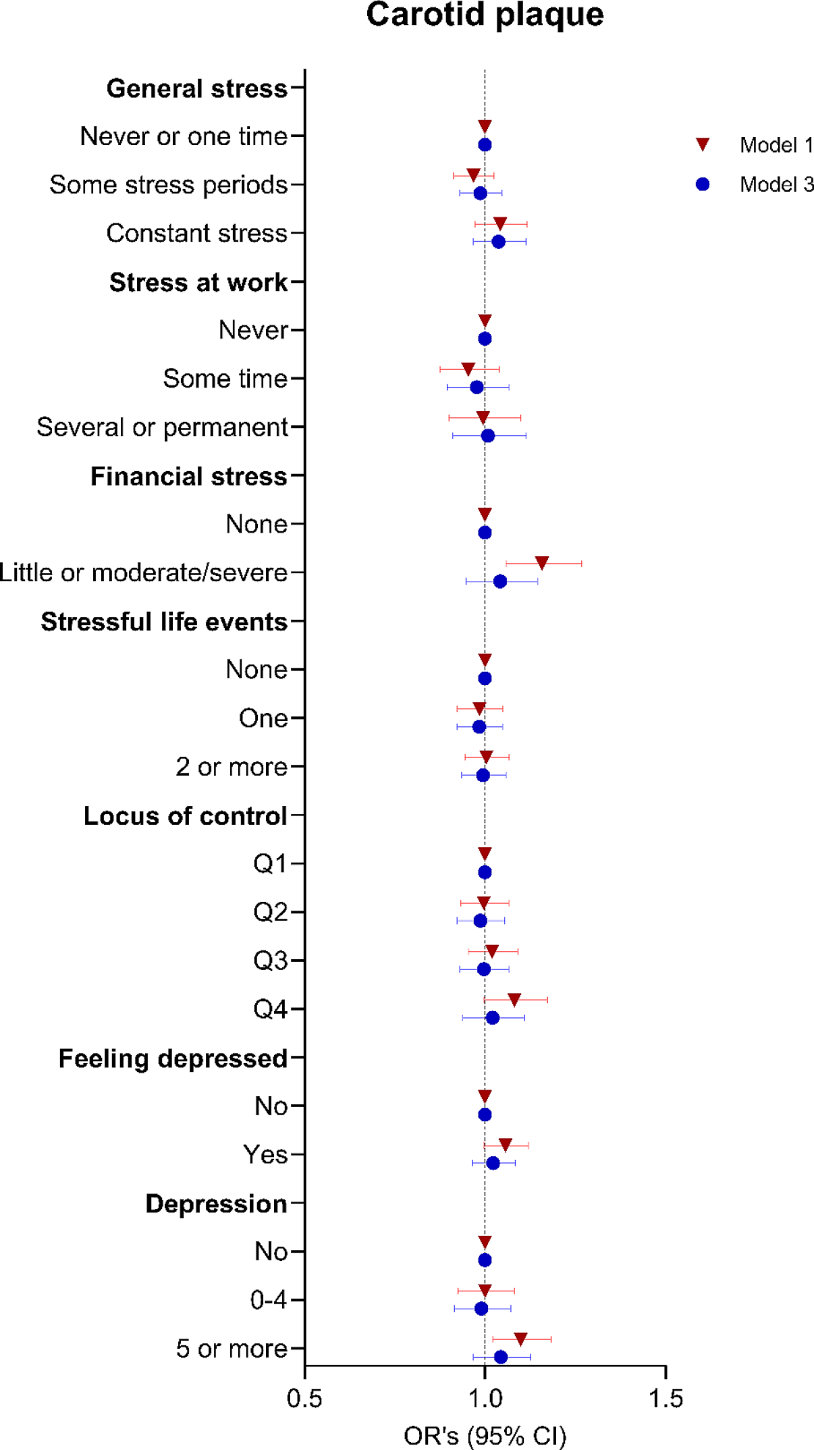
Psychological factors and carotid atherosclerosis The logistic regression model shows the odds ratios of the different psychological factors on any carotid plaques. Model 1 adjusted for sex, age, and site; Model 3 adjusted for model 1 plus educational level, marital status, alcohol, smoking, BMI, diet, physical activity, and sleep

Associations of psychological factors with carotid atherosclerosis are presented in Figure. 2 (detailed data in Supplementary Table 3). Generally, the results were comparable to those observed for coronary atherosclerosis. Thus, there was some evidence for associations between financial stress and depression with carotid plaques. However, these associations were attenuated and not statistically significant in model 3.

### Psychological factors and cardiovascular health assessed by Life’s Essential 8

Table 2 presents the results of the linear regression analyses between psychological factors with LE8. The results show an inverse association between most of the psychological factors with the total LE8 score as well as with the health behavior and factor scores. For instance, experiencing constant general stress, as compared to never/one time, was associated with lower LE8 score (β = -0.090, *p* < 0.001), which reflects a lower score in both health behaviors (β = -0.116 *p* < 0.001) and health factors (β = -0.039 *p* < 0.001). In addition, Supplementary Tables 4 and 5 show the associations of psychological factors with the 8 individual components of LE8 behaviors and factors, respectively. Generally, higher scores in stress, locus of control or depression were associated with worse scores in most of the components of LE8, and the results when adjusting for model 2 and model 3 were quite comparable. In accordance with Table 2, the associations of psychological factors were generally stronger for LE8 behaviors than for LE8 factors.

**Table 2:** Linear regression to examine the relationship of psychological factors with total, behavior, and factor scores of Life’s Essential 8

	Life’s Essential 8 (0-100)					
	Total score		Behaviors		Factors	
	β	*p*	β	*p*	β	*p*
**General stress**						
Never or one time	Reference		Reference		Reference	
Some stress periods	-0.003	0.672	-0.009	0.173	0.002	0.731
Constant stress	-0.090	< 0.001	-0.116	< 0.001	-0.039	< 0.001
**Stress at work**						
Never	Reference		Reference		Reference	
Some time	0.012	0.214	-0.006	0.547	0.018	0.057
Several or permanent	-0.033	< 0.001	-0.064	< 0.001	-0.002	0.809
**Financial stress**						
None	Reference		Reference		Reference	
Little or moderate/severe	-0.104	< 0.001	-0.125	< 0.001	-0.051	< 0.001
**Stressful life events**						
None	Reference		Reference		Reference	
1	-0.024	< 0.001	-0.023	< 0.001	-0.016	0.018
2 or more	-0.050	< 0.001	-0.056	< 0.001	-0.027	< 0.001
**Locus of control**						
Q1 (more internal)	Reference		Reference		Reference	
Q2	-0.028	< 0.001	-0.030	< 0.001	-0.016	0.022
Q3	-0.064	< 0.001	-0.071	< 0.001	-0.035	< 0.001
Q4 (less internal)	-0.128	< 0.001	-0.130	< 0.001	-0.079	< 0.001
**Feeling depressed**						
No	Reference		Reference		Reference	
Yes	-0.072	< 0.001	-0.106	< 0.001	-0.023	< 0.001
**Depression**						
No	Reference		Reference		Reference	
0–4 items	0.001	0.846	-0.029	< 0.001	0.020	< 0.001
5 items or more	-0.109	< 0.001	-0.131	< 0.001	-0.053	< 0.001

β: Standardized β-Coefficient (one unit is equal to a standard deviation change in the Life’s Essential 8); p: P value. Associations were adjusted for sex, age, site, education level, and marital status

## Discussion

This large population-based study investigated the association of psychological factors with coronary and carotid atherosclerosis as well as cardiovascular health measured by the LE8 concept. The results showed an association of financial stress and locus of control with coronary atherosclerosis, before adjusting for socioeconomic and important lifestyle variables. After adjustment (model 3), general stress, stress at work, financial stress, stressful life events, locus of control, feeling depressed and depression were not statistically significantly associated with an increased risk of atherosclerosis in the coronary arteries. However, an inverse association was observed between these psychological factors and the LE8 total score, as well as the LE8 behaviors and the LE8 factors scores.

Although some previous studies have established a link between psychological factors and cardiovascular disease or health [15, 32–35], few have examined the associations between psychological factors and atherosclerosis and these have reported conflicting results. For instance, in consonance with our results, a cross-sectional study of 1,849 middle-aged individuals in the United States found no significant association between perceived stress and CAC [36]. Conversely, in a study involving 5,140 adult participants without prior cardiovascular disease, a correlation between perceived stress and CIMT was observed, particularly among unemployed individuals [37]. In the present study, we did not identify a significant interaction between unemployment or educational status and psychological factors in relation to atherosclerosis or cardiovascular health. Nevertheless, further research is warranted to explore whether socioeconomic status modify the association between psychological factors and atherosclerosis.

Regarding general stress, a study involving a relatively small sample (*n* = 150) of managers and office workers found no evidence of an association between higher global stress levels and the presence of plaques in the carotid arteries [38]. These results are comparable to ours which reported no evidence of a positive association between general stress and atherosclerosis in a large sample of middle-aged individuals. Previous cross-sectional studies have reported no associations between work-related stress or job strain with CAC [36, 39, 40] which agrees with our findings. However, there was some evidence of a link between greater depressive symptoms and CAC in a previous study of middle-aged men and women [36]. In addition, a meta-analysis including 15 observational studies and 32,884 participants found that diagnosed depression was weakly, yet statistically significantly associated with a higher CAC scores. Nonetheless, depressive symptoms did not show a statistically significant association [41]. To summarize, the findings of these previous cross-sectional studies generally support our findings suggesting that there is little evidence to support strong and robust associations of a wide range of psychological factors with atherosclerosis. However, our study is considerably larger than most previously published studies which would have enabled detection of also relatively weak associations.

Interestingly, the few associations between psychological factors and atherosclerosis that were statistically significant in model 1 (adjusted for age, sex, and site) were attenuated after adjustments for socioeconomic factors and health behaviors. However, our study proved a consistent association between psychological factors and LE8, a novel metric created by the American Heart Association to monitor cardiovascular health [28]. Thus, although this study does not indicate a direct contribution of psychological factors on atherosclerosis, the potential indirect association between psychological factors and atherosclerosis may be relevant via modification of health behaviors and health factors. In this sense, we observed that psychological factors (i.e., general stress, stress at work, financial stress, stressful life events, locus of control, feeling depressed and depression) were generally associated with worse health behaviors (diet, physical activity, smoking, sleep) and factors (BMI, lipid level, glucose, and blood pressure) which aligns with previous literature and may be highly relevant considering the strong association between LE8 and later CVD [42–45]. Thus, the above-mentioned association between psychological factors and health behaviors and factors may be a relevant mechanism that could, at least partly, mediate a potential longitudinal association between psychological factors and atherosclerosis. However, only few studies have examined longitudinal associations of psychological factors in relation with later atherosclerosis and these studies have been relatively small (*n* = 149–311) [46, 47]. Thus, although our study provide evidence for a lack of direct associations (i.e., independent of socioeconomic variables and cardiovascular risk factors) between a wide range of psychological factors and atherosclerosis cross-sectionally (i.e., independent of cardiovascular risk behaviors), further studies are needed to examine the longitudinal influence of such psychological factors on atherosclerosis development and progression. Regarding sex, we did not find a clear interaction for the association between psychological factors and atherosclerosis or cardiovascular health. Previous studies have often lacked sex stratification, and among those that did, no significant differences were observed between groups, which is consistent with our findings. Nevertheless, we identified one study reporting significant differences in the association of social exposure variables with biomarkers of coronary heart disease. Specifically, in women, passive work was associated with diastolic and systolic hypertension [39]. More research is needed to clarify the different contribution of sex in the association of psychological factors and atherosclerosis.

The study has several important strengths including the population-based sampling and the sample size which is larger than most comparable studies [36, 38–40, 46, 47]. Furthermore, the use of high-quality imaging (CCTA) to measure subclinical coronary atherosclerosis is another strength of the study. To our knowledge, this is the first time that has been studied the associations between psychological factors and CCTA stenosis. In addition, the calculation of the LE8 score fulfils the American Heart Association criteria by incorporating 8 components and employing objective and standardized methods to assess physical activity (accelerometry), BMI, blood lipids, blood glucose, and blood pressure [29]. However, smoking, diet, and sleep were self-reported and prone to certain recall bias although objective measures like polysomnography may be impractical for population-based studies.

Our study has also several limitations. For example, the observational and cross-sectional design preclude conclusions about causal relationships and thus further studies are needed. Furthermore, despite a relatively high participation rate, and like most studies, there was an underrepresentation of low socioeconomic areas which may somewhat limit the generalizability of the results. Given the ambitious nature of the SCAPIS study, which involved time-consuming assessments, it is possible that individuals with significantly worse levels in there might be an underrepresentation of people with a high degree of psychological factors may be underrepresented. In addition, information on different plaque phenotypes or plaque burden was not available. To conclude, while the findings presented here may be generalizable to a middle-aged population, they may not extend to younger individuals who have not yet developed a high degree of subclinical atherosclerosis.

## Conclusion

This large population-based study showed no strong or consistent associations of psychological factors with coronary and carotid atherosclerosis, especially not after controlling for socioeconomic factors and health behaviors. However, the consistent associations of psychological factors with cardiovascular health (measured by LE8 behaviors and LE8 factors) may be relevant for participants’ future CVD risk. Thus, further studies are needed to elucidate the long-term effects of psychological factors on later atherosclerosis and cardiovascular health.

### Electronic supplementary material

Below is the link to the electronic supplementary material.

Supplementary Material 1

## Data Availability

The data underlying this article cannot be shared publicly due to legal reasons as well as the privacy of individuals who participated in the study. However, by contacting the study organization (www.scapis.org) or corresponding author, information will be provided regarding the procedures for accessing data following Swedish legislation.

## References

[1] Vaduganathan M, Mensah GA, Turco JV, Fuster V, Roth GA (2022). The Global Burden of Cardiovascular diseases and Risk: a compass for Future Health. J Am Coll Cardiol.

[2] Tsao CW, Aday AW, Almarzooq ZI, Anderson CAM, Arora P, Avery CL et al. Heart Disease and Stroke Statistics-2023 update: a Report from the American Heart Association. Circulation. 2023;147. 10.1161/CIR.0000000000001123 PMC12135016 36695182

[3] Bergström G, Persson M, Adiels M, Björnson E, Bonander C, Ahlström H (2021). Prevalence of subclinical coronary artery atherosclerosis in the General Population. Circulation.

[4] Lu SX, Wu TW, Chou CL, Cheng CF, Wang LY (2023). Combined effects of hypertension, hyperlipidemia, and diabetes mellitus on the presence and severity of carotid atherosclerosis in community-dwelling elders: a community-based study. J Chin Med Assoc.

[5] Cuspidi C, Sala C, Tadic M, Gherbesi E, Grassi G, Mancia G (2019). Pre-hypertension and subclinical carotid damage: a meta-analysis. J Hum Hypertens.

[6] Stein JH, Smith SS, Hansen KM, Korcarz CE, Piper ME, Fiore MC (2020). Longitudinal effects of smoking cessation on carotid artery atherosclerosis in contemporary smokers: the Wisconsin smokers Health Study. Atherosclerosis.

[7] Kianoush S, Yakoob MY, Al-Rifai M, DeFilippis AP, Bittencourt MS, Duncan BB et al. Associations of cigarette smoking with subclinical inflammation and atherosclerosis: ELSA-Brasil (the Brazilian Longitudinal Study of Adult Health). J Am Heart Assoc. 2017;6. 10.1161/JAHA.116.005088 PMC5669156 28647689

[8] Cuspidi C, Sala C, Tadic M, Rescaldani M, De Giorgi GA, Grassi G (2015). Untreated masked hypertension and carotid atherosclerosis: a meta-analysis. Blood Press.

[9] Osborne MT, Shin LM, Mehta NN, Pitman RK, Fayad ZA, Tawakol A (2020). Disentangling the links between psychosocial stress and Cardiovascular Disease. Circ Cardiovasc Imaging.

[10] Kivimäki M, Steptoe A (2018). Effects of stress on the development and progression of cardiovascular disease. Nat Rev Cardiol.

[11] Kelsey RM, Blascovich J, Tomaka J, Leitten CL, Schneider TR, Wiens S (1999). Cardiovascular reactivity and adaptation to recurrent psychological stress: effects of prior task exposure. Psychophysiology.

[12] Moberg E, Kollind M, Lins PE, Adamson U (1994). Acute mental stress impairs insulin sensitivity in IDDM patients. Diabetologia.

[13] Ghiadoni L, Donald AE, Cropley M, Mullen MJ, Oakley G, Taylor M (2000). Mental stress induces transient endothelial dysfunction in humans. Circulation.

[14] Lechner K, von Schacky C, McKenzie AL, Worm N, Nixdorff U, Lechner B (2020). Lifestyle factors and high-risk atherosclerosis: pathways and mechanisms beyond traditional risk factors. Eur J Prev Cardiol.

[15] Rosengren A, Hawken S, Ôunpuu S, Sliwa PK, Zubaid M, Almahmeed WA (2004). Association of psychosocial risk factors with risk of acute myocardial infarction in 11119 cases and 13648 controls from 52 countries (the INTERHEART study): case-control study. Lancet.

[16] Hernandez R, Allen NB, Liu K, Stamler J, Reid KJ, Zee PC (2014). Association of depressive symptoms, trait anxiety, and perceived stress with subclinical atherosclerosis: results from the Chicago healthy aging study (CHAS). Prev Med (Baltim).

[17] Shah BM, Shah S, Kandula NR, Gadgil MD, Kanaya AM (2016). Psychosocial Factors Associated with subclinical atherosclerosis in South asians: the MASALA Study. J Immigr Minor Health.

[18] Khan A, Palka J, Joshi PH, Khera A, Brown ES (2020). Association of depressive symptom severity with coronary artery calcium: the Dallas heart study. J Affect Disord.

[19] Seldenrijk A, Vogelzangs N, van Hout HPJ, van Marwijk HWJ, Diamant M, Penninx BWJH (2010). Depressive and anxiety disorders and risk of subclinical atherosclerosis findings from the Netherlands Study of Depression and anxiety (NESDA). J Psychosom Res.

[20] Murgia A, Balestrieri A, Crivelli P, Suri JS, Conti M, Cademartiri F (2020). Cardiac computed tomography radiomics: an emerging tool for the non-invasive assessment of coronary atherosclerosis. Cardiovasc Diagnosis Therapy.

[21] Han D, Hartaigh B, Gransar H, Lee JH, Rizvi A, Baskaran L (2018). Incremental prognostic value of coronary computed tomography angiography over coronary calcium scoring for major adverse cardiac events in elderly asymptomatic individuals. Eur Heart J Cardiovasc Imaging.

[22] Bergström G, Berglund G, Blomberg A, Brandberg J, Engström G, Engvall J (2015). The Swedish CArdioPulmonary BioImage Study: objectives and design. J Intern Med.

[23] Kristensen TS, Hannerz H, Høgh A, Borg V (2005). The Copenhagen Psychosocial Questionnaire–a tool for the assessment and improvement of the psychosocial work environment. Scand J Work Environ Health.

[24] Raff GL, Chair, Abidov A, Achenbach S, Berman DS, Boxt LM (2009). SCCT guidelines for the interpretation and reporting of coronary computed tomographic angiography. J Cardiovasc Comput Tomogr.

[25] Agatston AS, Janowitz WR, Hildner FJ, Zusmer NR, Viamonte M, Detrano R (1990). Quantification of coronary artery calcium using ultrafast computed tomography. J Am Coll Cardiol.

[26] McCollough CH, Ulzheimer S, Halliburton SS, Shanneik K, White RD, Kalender WA (2007). Coronary artery calcium: a multi-institutional, multimanufacturer international standard for quantification at cardiac CT. Radiology.

[27] Touboul PJ, Hennerici MG, Meairs S, Adams H, Amarenco P, Bornstein N et al. Mannheim carotid intima-media thickness and plaque consensus (2004-2006-2011). An update on behalf of the advisory board of the 3rd, 4th and 5th watching the risk symposia, at the 13th, 15th and 20th European Stroke Conferences, Mannheim, Germany, 2004, Brussels, Belgium, 2006, and Hamburg, Germany, 2011. Cerebrovasc Dis. 2012;34:290–6. 10.1159/000343145 PMC3760791 23128470

[28] Lloyd-Jones DM, Allen NB, Anderson CAM, Black T, Brewer LC, Foraker RE (2022). Life’s essential 8: updating and enhancing the American Heart Association’s construct of Cardiovascular Health: a Presidential Advisory from the American Heart Association. Circulation.

[29] Herraiz-Adillo Á, Higueras-Fresnillo S, Ahlqvist VH, Berglind D, Syrjälä MB, Daka B et al. Life’s Essential 8 and Life’s Simple 7 in Relation to Coronary Atherosclerosis: Results From the Population-Based SCAPIS Project. Mayo Clin. 2023. 10.1016/j.mayocp.2023.03.023 37843486

[30] Cerwinske LA, Rasmussen HE, Lipson S, Volgman AS, Tangney CC (2017). Evaluation of a dietary screener: the Mediterranean Eating Pattern for americans tool. J Hum Nutr Diet.

[31] Ekblom-Bak E, Börjesson M, Bergman F, Bergström G, Dahlin-Almevall A, Drake I et al. Accelerometer derived physical activity patterns in 27.890 middle-aged adults: the SCAPIS cohort study. Scand J Med Sci Sports. 2022;32. 10.1111/sms.14131 PMC9302631 35080270

[32] Sun Y, Zhang H, Wang B, Chen C, Chen Y, Chen Y (2022). Joint exposure to positive affect, life satisfaction, broad depression, and neuroticism and risk of cardiovascular diseases: a prospective cohort study. Atherosclerosis.

[33] Moretti Anfossi C, Ahumada Muñoz M, Tobar Fredes C, Pérez Rojas F, Ross J, Head J (2022). Work exposures and Development of Cardiovascular diseases: a systematic review. Annals Work Exposures Health.

[34] Araki A, Ito H (2013). Psychological risk factors for the development of stroke in the Elderly. J Neurol Neurophysiol.

[35] Mathews L, Ogunmoroti O, Nasir K, Blumenthal RS, Utuama OA, Rouseff M (2018). Psychological Factors and Their Association with Ideal Cardiovascular Health among women and men. J Womens Health.

[36] Rozanski A, Gransar H, Kubzansky LD, Wong N, Shaw L, Miranda-Peats R (2011). Do psychological risk factors predict the presence of coronary atherosclerosis?. Psychosom Med.

[37] Wiernik E, Lemogne C, Thomas F, Perier MC, Guibout C, Nabi H (2016). Perceived stress, common carotid intima media thickness and occupational status: the Paris prospective study III. Int J Cardiol.

[38] Bugajska J, Widerszal-Bazyl M, Radkiewicz P, Pasierski T, Szulczyk GA, Za̧bek J (2008). Perceived work-related stress and early atherosclerotic changes in healthy employees. Int Arch Occup Environ Health.

[39] Söderberg M, Eriksson H, Torén K, Bergström G, Andersson E, Rosengren A (2022). Psychosocial job conditions and biomarkers of cardiovascular disease: a cross-sectional study in the Swedish CArdioPulmonary bioImage study (SCAPIS). Scand J Public Health.

[40] Eriksson H, Torén K, Rosengren A, Andersson E, Söderberg M. Psychosocial job exposure and risk of coronary artery calcification. PLoS ONE. 2021;16. 10.1371/journal.pone.0252192 PMC8148350 34033665

[41] Lin S, Zhang H, Ma A (2018). The association between depression and coronary artery calcification: a meta-analysis of observational studies. J Affect Disord.

[42] Li X (2023). Life’s Essential 8, Genetic Susceptibility, and Incident Cardiovascular Disease: A Prospective Study. Arterioscler Thromb Vasc Biol.

[43] Rempakos A, Prescott B, Mitchell GF, Vasan RS, Xanthakis V. Association of life’s essential 8 with cardiovascular disease and mortality: the framingham heart study. J Am Heart Assoc. 2023 Dec 5;12(23):e030764. 10.1161/JAHA.123.030764 PMC10727315 38014669

[44] Jin C, Li J, Liu F, Li X, Hui Y, Chen S, Li F, Wang G, Liang F, Lu X, Wu S, Gu D. Life’s Essential 8 and 10-year and lifetime risk of atherosclerotic cardiovascular disease in China. Am J Prev Med. 2023 Jun;64(6):927-935 10.1016/j.amepre.2023.01.009 36813641

[45] Isiozor NM, Laukkanen JA, Voutilainen A, Bensenor IM, Kunutsor SK. Life’s Essential 8 is associated with atherosclerotic cardiovascular disease but not venous thromboembolism in men: a prospective cohort study. Ann Med. 2023 Dec;55(1):2233894. 10.1080/07853890.2023.2233894 PMC10353322 37459575

[46] Juonala M, Pulkki-Råback L, Elovainio M, Hakulinen C, Magnussen CG, Sabin MA (2016). Childhood psychosocial factors and coronary artery calcification in Adulthood: the Cardiovascular Risk in Young finns Study. JAMA Pediatr.

[47] Low CA, Matthews KA, Kuller LH, Edmundowicz D (2011). Psychosocial predictors of coronary artery calcification progression in Postmenopausal Women. Psychosom Med.

