# Supplementary material for: Associations of psychological factors with atherosclerosis and cardiovascular health in middle-age: the population-based Swedish CArdioPulmonary bioImage study (SCAPIS)
Source: BMC Public Health. 2024 May 30;24:1455. doi: 10.1186/s12889-024-18924-w (PMC11140911; doi:10.1186/s12889-024-18924-w)
Supplement: Supplementary file 1 — Supplementary Material 1 [file 12889_2024_18924_MOESM1_ESM.docx]

**Associations of psychological factors with**

**atherosclerosis and cardiovascular health in middle-age:**

**the population-based Swedish CArdioPulmonary bioImage Study (SCAPIS)**

Sara Higueras-Fresnillo^1,2^, Ángel Herraiz-Adillo^1^, Viktor H. Ahlqvist^3^, Robin Öberg^1^, Cecilia Lenander^4^, Patrik Wennberg^5^, Josefin Wångdahl^6,7^, Daniel Berglind^3,8^, Bledar Daka^9^, Carl Johan Östgren^1,10^, Karin Rådholm^1,11^, Pontus Henriksson^1^.

1. Department of Health, Medicine and Caring Sciences, Linköping University, Linköping, Sweden
2. Department of Preventive Medicine and Public Health, Universidad Autónoma de Madrid, Madrid, Spain
3. Department of Global Public Health, Karolinska Institutet, Stockholm, Sweden
4. Department of Clinical Sciences in Malmö, Centre for Primary Health Care Research, Lund University, Lund, Sweden
5. Department of Public Health and Clinical Medicine, Umeå University, Umeå, Sweden
6. Aging Research Center, Karolinska Institutet & Stockholm University, Stockholm, Sweden
7. Department of Public Health and Caring Sciences, Uppsala, Uppsala University
8. Centre for Epidemiology and Community Medicine, Region Stockholm, Stockholm, Sweden
9. Family medicine, School of Public Health and Community Medicine, Institute of Medicine, Sahlgrenska Academy, University of Gothenburg
10. Centre of Medical Image Science and Visualization (CMIV), Linköping University, Linköping, Sweden
11. The George Institute for Global Health, University of New South Wales, Sydney, Australia

Corresponding author: [sara.higueras@uam.es](mailto:sara.higueras@uam.es)

**Supplementary material**


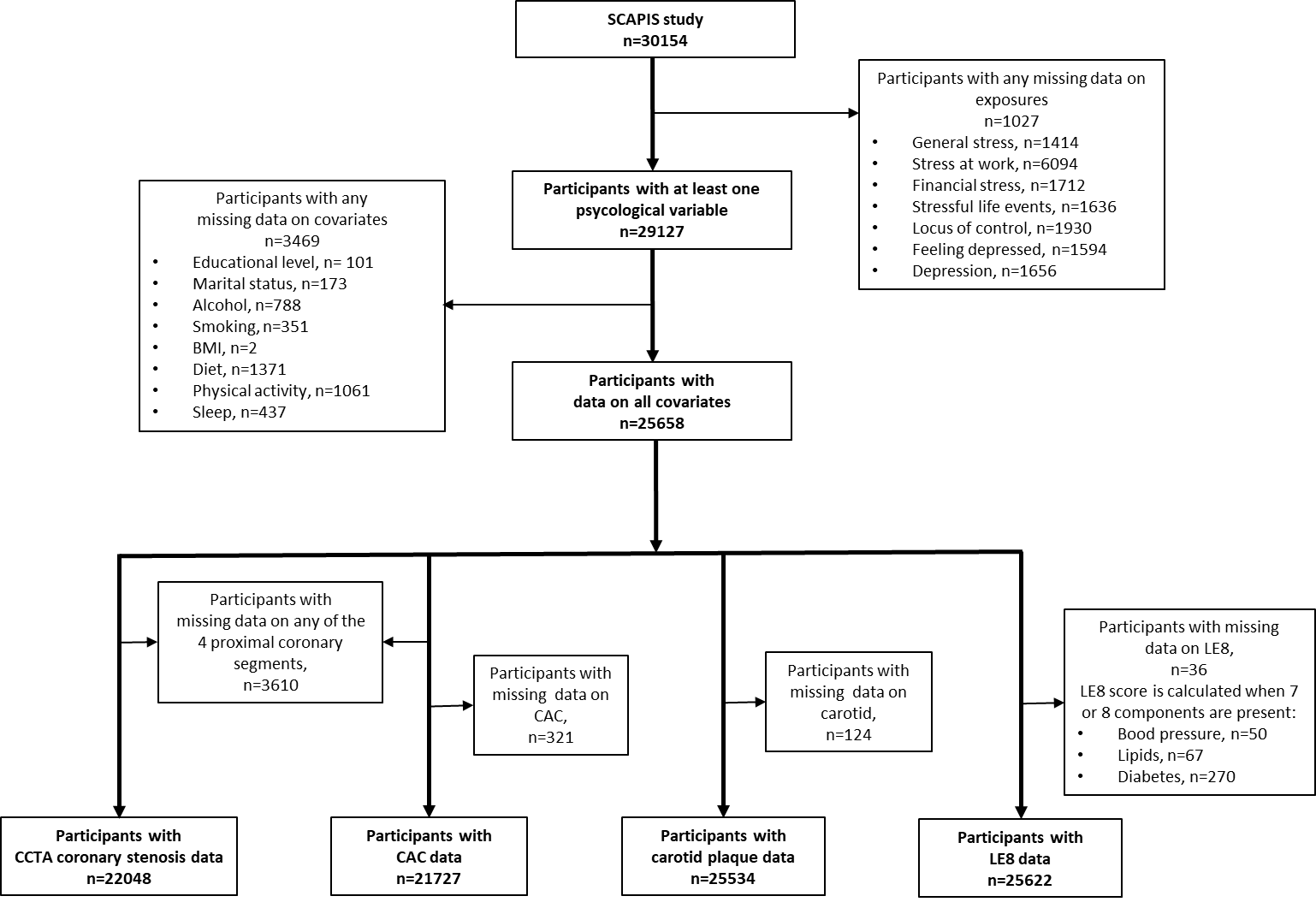


**Supplementary Figure 1.** Flow chart of the study.
SCAPIS: Swedish CArdioPulmonary bioImage Study; CCTA: coronary computed tomographic angiography; CAC: coronary artery calcium; MVPA: moderate-vigorous physical activity.

**Supplementary Table 1.** Logistic regression to estimate the associations between psychological factors and the odds of any CCTA stenosis and CAC scores ≥1.

|  | | | | | |  | | |
| --- | --- | --- | --- | --- | --- | --- | --- | --- |
|  | | **CCTA** | | |  | **CAC** | | |
|  | | **Model 1**  **ORs (CI 95%)** | **Model 2**  **ORs (CI 95%)** | **Model 3**  **ORs (CI 95%)** |  | **Model 1**  **ORs (CI 95%)** | **Model 2**  **ORs (CI 95%)** | **Model 3**  **ORs (CI 95%)** |
| **General stress** | | n=21908 | n=21908 | n=21908 |  | n=21591 | n=21591 | n=21591 |
| Never or one time | | Reference | Reference | Reference |  | Reference | Reference | Reference |
| Some stress periods | | 0.974 (0.914-1.039) | 0.994 (0.932-1.061) | 0.996 (0.933-1.064) |  | 0.951 (0.891-1.016) | 0.972 (0.910-1.037) | 0.972 (0.910-1.039) |
| Constant stress | | 1.044 (0.965-1.130) | 1.057 (0.977-1.144) | 1.012 (0.933-1.097) |  | 1.016 (0.949-1.114) | 1.041 (0.960-1.128) | 0.992 (0.910-1.077) |
| **Stress at work** | | n=18804 | n=18804 | n=18804 |  | n=18551 | n=18551 | n=18551 |
| Never | | Reference | Reference | Reference |  | Reference | Reference | Reference |
| Some time | | 0.982 (0.893-1.080) | 0.999 (0.908-1.100) | 1.015 (0.922-1.119) |  | 0.977 (0.887-1.076) | 0.994 (0.902-1.095) | 1.009 (0.915-1.113) |
| Several or permanent | | 1.011 (0.903-1.130) | 1.029 (0.920-1.152) | 1.025 (0.915-1.149) |  | 0.991 (0.884-1.111) | 1.010 (0.900-1.132) | 1.001 (0.891-1.124) |
| **Financial stress** | n=21647 | | n=21647 | n=21647 |  | n=21334 | n=21334 | n=21334 |
| None | | Reference | Reference | Reference |  | Reference | Reference | Reference |
| Little or moderate/severe | | **1.149 (1.035-1.276)** | 1.093 (0.982-1.216) | 0.982 (0.880-1.095) |  | **1.173 (1.054-1.305)** | **1.115 (1.000-1.243)** | 0.999 (0.894-1.116) |
| **Stressful life events** | n=21832 | | n=21832 | n=21832 |  | n=21513 | n=21513 | n=21513 |
| None | | Reference | Reference | Reference |  | Reference | Reference | Reference |
| 1 | | 1.009 (0.940-1.084) | 1.012 (0.943-1.087) | 1.002 (0.932-1.076) |  | 1.018 (0.948-1.095) | 1.022 (0.950-1.098) | 1.011 (0.940-1.087) |
| 2 or more | | 1.060 (0.990-1.136) | 1.060 (0.989-1.136) | 1.034 (0.964-1.108) |  | 1.053 (0.982-1.129) | 1.054 (0.983-1.130) | 1.028 (0.958-1.103) |
| **Locus of control** | n=21646 | | n=21646 | n=21646 |  | n=21334 | n=21334 | n=21334 |
| Q1 (more internal) | | Reference | Reference | Reference |  | Reference | Reference | Reference |
| Q2 | | 0.980 (0.909-1.056) | 0.974 (0.904-1.050) | 0.958 (0.888-1.034) |  | 0.995 (0.922-1.074) | 0.990 (0.917-1.068) | 0.973 (0.901-1.051) |
| Q3 | | 1.066 (0.988-1.149) | 1.049 (0.972-1.131) | 1.016 (0.941-1.098) |  | 1.042 (0.965-1.125) | 1.026 (0.950-1.108) | 0.993 (0.918-1.074) |
| Q4 (less internal) | | **1.208 (1.104-1.324)** | **1.171 (1.068-1.285)** | 1.080 (0.983-1.187) |  | **1.175 (1.070-1.289)** | **1.138 (1.036-1.250)** | 1.045 (0.950-1.150) |
| **Feeling depressed** | n=21818 | | n=21818 | n=21818 |  | n=21500 | n=21500 | n=21500 |
| No | | Reference | Reference | Reference |  | Reference | Reference | Reference |
| Yes | | **1.124 (1.052-1.201)** | **1.114 (1.042-1.191)** | 1.068 (0.998-1.142) |  | **1.120 (1.047-1.198)** | **1.110 (1.037-1.188)** | 1.063 (0.992-1.138) |
| **Depression** | | n=21774 | n=21774 | n=21774 |  | n=21457 | n=21457 | n=21457 |
| No | | Reference | Reference | Reference |  | Reference | Reference | Reference |
| 0-4 items | | 1.055 (0.965-1.153) | 1.052 (0.963-1.150) | 1.052 (0.962-1.151) |  | 1.084 (0.991-1.186) | 1.082 (0.989-1.184) | 1.082 (0.988-1.185) |
| 5 items or more | | **1.192 (1.095-1.298)** | **1.176 (1.079-1.281)** | 1.083 (0.992-1.182) |  | **1.154 (1.058-1.259)** | **1.138 (1.042-1.242)** | 1.045 (0.956-1.143) |

CCTA: coronary computed tomographic angiography, CAC: coronary artery calcium. Model 1: adjusted for sex, age, site; Model 2: adjusted for sex, age, site, education level and marital status; Model 3: adjusted for sex, age, site, education level, marital status, alcohol, smoking, body mass index, diet, physical activity, and sleep.

**Supplementary Table 2**. Logistic regression to estimate the associations between stressful life events <12 months ago or ≥12 months ago and any CCTA stenosis.

|  | **CCTA** | | |
| --- | --- | --- | --- |
|  | **Model 1**  **ORs (CI 95%)** | **Model 2**  **ORs (CI 95%)** | **Model 3**  **ORs (CI 95%)** |
| Stressful life events, <12 months ago  (Main analysis) | n=21832 | n=21832 | n=21832 |
| None | Reference | Reference | Reference |
| 1 | 1.009 (0.940-1.084) | 1.012 (0.943-1.087) | 1.002 (0.932-1.076) |
| 2 or more | 1.060 (0.990-1.136) | 1.060 (0.989-1.136) | 1.034 (0.964-1.108) |
| Stressful life events, ≥12 months ago | n=21832 | n=21832 | n=21832 |
| None | Reference | Reference | Reference |
| 1 | 1.013 (0.923-1.113) | 1.012 (0.921-1.112) | 1.013 (0.921-1.114) |
| 2 or more | 1.020 (0.946-1.099) | 1.010 (0.936-1.089) | 0.996 (0.923-1.075) |

CCTA: coronary computed tomographic angiography. Model 1: adjusted for sex, age and site; Model 2: adjusted for sex, age, site, education level and marital status; Model 3: adjusted for sex, age, site, education level, marital status, alcohol, smoking, body mass index, diet, physical activity and sleep.

**Supplementary Table 3.** Logistic regression to estimate the associations between psychological factors and any carotid plaque.

|  | | | |
| --- | --- | --- | --- |
|  | **Carotid plaque (0 vs. any carotid plaque)** | | |
|  | **Model 1**  **ORs (CI 95%)** | **Model 2**  **ORs (CI 95%)** | **Model 3**  **ORs (CI 95%)** |
| General stress | n=25357 | n=25357 | n=25357 |
| Never or one time | Reference | Reference | Reference |
| Some stress periods | 0.968 (0.914-1.025) | 0.985 (0.929-1.043) | 0.987 (0.931-1.046) |
| Constant stress | 1.042 (0.973-1.117) | 1.065 (0.981-1.127) | 1.038 (0.968-1.114) |
| Stress at work | n=21501 | n=21501 | n=21501 |
| Never | Reference | Reference | Reference |
| Some time | 0.954 (0.876-1.040) | 0.968 (0.888-1.056) | 0.977 (0.896-1.066) |
| Several or permanent | 0.995 (0.900-1.099) | 1.009 (0.913-1.115) | 1.008 (0.911-1.115) |
| Financial stress | n=25026 | n=25026 | n=25026 |
| Little or none | Reference | Reference | Reference |
| Moderate or severe | **1.158** (**1.058-1.268**) | **1.103** (**1.005-1.221**) | 1.042 (0.948-1.145) |
| Stressful life events | n=25242 | n=25242 | n=25242 |
| None | Reference | Reference | Reference |
| 1 | 0.985 (0.924-1.049) | 0.987 (0.927-1.052) | 0.984 (0.923-1.048) |
| 2 or more | 1.004 (0.945-1.067) | 1.004 (0.945-1.067) | 0.995 (0.936-1.058) |
| Locus of control | n=25009 | n=25009 | n=25009 |
| Q1 (more internal) | Reference | Reference | Reference |
| Q2 | 0.977 (0.933-1.066) | 0.992 (0.928-1.060) | 0.987 (0.923-1.055) |
| Q3 | 1.020 (0.954-1.091) | 1.006 (0.940-1.076) | 0.997 (0.931-1.066) |
| Q4 (less internal) | 1.082 (0.998-1.175) | 1.051 (0.969-1.140) | 1.021 (0.939-1.109) |
| Feeling depressed | n=25241 | n=25241 | n=25241 |
| No | Reference | Reference | Reference |
| Yes | 1.057 (0.998-1.120) | 1.045 (0.986-1.108) | 1.023 (0.965-1.085) |
| Depression | n=25189 | n=25189 | n=25189 |
| No | Reference | Reference | Reference |
| 0-4 items | 1.000 (0.925-1.081) | 0.995 (0.921-1.076) | 0.990 (0.915-1.071) |
| 5 items or more | **1.099** (**1.021-1.183**) | **1.081** (**1.003 -1.164**) | 1.044 (0.968-1.126) |

Model 1: adjusted for sex, age and site; Model 2: adjusted for sex, age, site, education level and marital status; Model 3: adjusted for sex, age, site, education level, marital status, alcohol, smoking, body mass index, diet, physical activity and sleep.

| **Supplementary Table 4**. Linear regression to examine the relationship between psychological factors and cardiovascular health  behaviors (by Life’s Essential 8). | | | | | | | | |
| --- | --- | --- | --- | --- | --- | --- | --- | --- |
|  | **LE8 Diet** | | **LE8 Physical activity** | | **LE8 Smoking status** | | **LE8 Sleep** | |
|  | **Model 2** | **Model 3** | **Model 2** | **Model 3** | **Model 2** | **Model 3** | **Model 2** | **Model 3** |
|  | β (p) | β (p) | β (p) | β (p) | β (p) | β (p) | β (p) | β (p) |
| **General stress** |  |  |  |  |  |  |  |  |
| Never or one time | Reference |  | Reference |  | Reference |  | Reference |  |
| Some stress periods | -0.007 (0.332) | -0.004 (0.589) | 0.010 (0.164) | 0.006 (0.384) | 0.004 (0.600) | 0.011 (0.103) | **-0.023 (<0.001)** | **-0.026 (<0.001)** |
| Constant stress | **-0.016 (0.016)** | -0.008 (0.225) | **-0.023 (<0.001)** | **-0.018 (0.008)** | **-0.027 (<0.001)** | **-0.014 (0.032)** | **-0.183 (<0.001)** | **-0.177 (<0.001)** |
| **Stress at work** |  |  |  |  |  |  |  |  |
| Never | Reference |  | Reference |  | Reference |  | Reference |  |
| Some time | **-0.030 (0.002)** | **-0.030 (0.002)** | **0.042 (<0.001)** | **0.039 (<0.001)** | **0.032 (<0.001)** | **0.036 (<0.001)** | **-0.052 (<0.001)** | **-0.054 (<0.001)** |
| Several or permanent | **-0.025 (0.010)** | **-0.025 (0.010)** | 0.018 (0.079) | 0.017 (0.084) | 0.005 (0.579) | 0.010 (0.300) | **-0.128 (<0.001)** | **-0.127 (<0.001)** |
| **Financial stress** |  |  |  |  |  |  |  |  |
| Little or none | Reference |  | Reference |  | Reference |  | Reference |  |
| Moderate or severe | 0.008 (0.177) | 0.012 (0.053) | **-0.040 (<0.001)** | **-0.020 (0.002)** | **-0.098 (<0.001)** | **-0.102 (<0.001)** | **-0.111 (<0.001)** | **-0.097 (<0.001)** |
| **Stressful life events** |  |  |  |  |  |  |  |  |
| None | Reference |  | Reference |  | Reference |  | Reference |  |
| 1 | -0.011 (0.113) | -0.009 (0.156) | -0.012 (0.084) | -0.010 (0.154) | -0.009 (0.189) | -0.006 (0.335) | **-0.024 (<0.001)** | **-0.021 (0.002)** |
| 2 or more | -0.003 (0.625) | 0.000 (0.946) | **-0.015 (0.031)** | -0.010 (0.165) | **-0.023 (<0.001)** | **-0.020 (0.004)** | **-0.076 (<0.001)** | **-0.070 (<0.001)** |
| **Locus of control** |  |  |  |  |  |  |  |  |
| Q1 (more internal) | Reference |  | Reference |  | Reference |  | Reference |  |
| Q2 | **-0.023 (0.001)** | **-0.020 (0.004)** | -0.004 (0.535) | 0.000 (0.989) | -0.012 (0.083) | -0.009 (0.213) | **-0.028 (<0.001)** | **-0.024 (<0.001)** |
| Q3 | **-0.043 (<0.001)** | **-0.039 (<0.001)** | **-0.017 (0.016)** | -0.010 (0.172) | **-0.023 (0.001)** | **-0.016 (0.017)** | **-0.078 (<0.001)** | **-0.072 (<0.001)** |
| Q4 (less internal) | **-0.027 (<0.001)** | **-0.022 (0.001)** | **-0.050 (<0.001)** | **-0.033 (<0.001)** | **-0.055 (<0.001)** | **-0.052 (<0.001)** | **-0.152 (<0.001)** | **-0.137 (<0.001)** |
| **Feeling depressed** |  |  |  |  |  |  |  |  |
| No | Reference |  | Reference |  | Reference |  | Reference |  |
| Yes | 0.009 (0.148) | **0.012 (0.049)** | **-0.034 (<0.001)** | **-0.025 (<0.001)** | **-0.051 (<0.001)** | **-0.048 (<0.001)** | **-0.141 (<0.001)** | **-0.133 (<0.001)** |
| **Depression** |  |  |  |  |  |  |  |  |
| No | Reference |  | Reference |  | Reference |  | Reference |  |
| 0-4 items | 0.012 (0.051) | 0.011 (0.083) | -0.010 (0.114) | -0.010 (0.101) | -0.011 (0.074) | -0.011 (0.073) | **-0.050 (<0.001)** | **-0.050 (<0.001)** |
| 5 items or more | 0.001 (0.896) | 0.007 (0.261) | **-0.041 (<0.001)** | **-0.028 (<0.001)** | **-0.066 (<0.001)** | **-0.062 (<0.001)** | **-0.164 (<0.001)** | **-0.152 (<0.001)** |
| β: Standardized β-Coefficient (one unit is equal to a standard deviation change in the Life’s Essential 8.); LE8: Life’s Essential 8; p: P value.  Model 2: associations were adjusted for sex, age, site, education level and marital status.  Model 3: associations were adjusted for sex, age, site, education level, marital status, alcohol, BMI, diet (except for LE8 Diet), physical activity (except for LE8 Physical activity), smoke (except for LE8 Smoke status), and sleep (except for LE8 Sleep). | | | | | | | | |
| **Supplementary Table 5**. Linear regression to examine the relationship between psychological factors and cardiovascular health  factors (by Life’s Essential 8). | | | | | | | | |
|  | **LE8 BMI** | | **LE8 Lipids** | | **LE8 Blood glucose** | | **LE8 Blood pressure** | |
|  | **Model 2** | **Model 3** | **Model 2** | **Model 3** | **Model 2** | **Model 3** | **Model 2** | **Model 3** |
|  | β (p) | β (p) | β (p) | β (p) | β (p) | β (p) | β (p) | β (p) |
| **General stress** |  |  |  |  |  |  |  |  |
| Never or one time | Reference |  | Reference |  | Reference |  | Reference |  |
| Some stress periods | 0.012 (0.067) | **0.017 (0.012)** | -0.012 (0.069) | **-0.013 (0.045)** | 0.008 (0.215) | 0.005 (0.415) | 0.000 (0.983) | -0.001 (0.926) |
| Constant stress | **-0.047 (<0.001)** | **-0.032 (<0.001)** | **-0.020 (0.004)** | -0.009 (0.168) | **-0.017 (0.010)** | 0.003 (0.645) | **-0.016 (0.016)** | 0.002 (0.806) |
| **Stress at work** |  |  |  |  |  |  |  |  |
| Never | Reference |  | Reference |  | Reference |  | Reference |  |
| Some time | 0.017 (0.080) | **0.021 (0.029)** | -0.007 (0.471) | -0.011 (0.246) | 0.011 (0.245) | 0.007 (0.432) | **0.025 (0.010)** | **0.022 (0.013)** |
| Several or permanent | -0.009 (0.382) | 0.000 (0.979) | -0.010 (0.332) | -0.009 (0.337) | -0.005 (0.576) | -0.001 (0.944) | 0.015 (0.128) | 0.017 (0.062) |
| **Financial stress** |  |  |  |  |  |  |  |  |
| Little or none | Reference |  | Reference |  | Reference |  | Reference |  |
| Moderate or severe | **-0.068 (<0.001)** | **-0.061 (<0.001)** | -0.009 (0.144) | 0.006 (0.386) | **-0.040 (<0.001)** | **-0.014 (0.026)** | **-0.018 (0.004)** | -0.003 (0.585) |
| **Stressful life events** |  |  |  |  |  |  |  |  |
| None | Reference |  | Reference |  | Reference |  | Reference |  |
| 1 | **-0.016 (0.019)** | -0.011 (0.113) | **-0.021 (0.003)** | **-0.017 (0.011)** | -0.006 (0.337) | -0.001 (0.893) | 0.003 (0.664) | 0.008 (0.222) |
| 2 or more | **-0.038 (<0.001)** | **-0.031 (<0.001)** | -0.014 (0.046) | -0.007 (0.318) | -0.011 (0.104) | 0.002 (0.793) | -0.008 (0.258) | 0.003 (0.688) |
| **Locus of control** |  |  |  |  |  |  |  |  |
| Q1 (more internal) | Reference |  | Reference |  | Reference |  | Reference |  |
| Q2 | **-0.021 (0.003)** | **-0.014 (0.039)** | -0.014 (0.054) | -0.008 (0.254) | -0.013 (0.061) | -0.004 (0.575) | 0.005 (0.444) | **0.013 (0.043)** |
| Q3 | **-0.042 (<0.001)** | **-0.030 (<0.001)** | **-0.015 (0.029)** | -0.006 (0.397) | **-0.029 (<0.001)** | -0.012 (0.065) | -0.007 (0.319) | 0.007 (0.314) |
| Q4 (less internal) | **-0.097 (<0.001)** | **-0.083 (<0.001)** | **-0.030 (<0.001)** | -0.011 (0.105) | **-0.048 (<0.011)** | -0.012 (0.067) | **-0.031 (<0.001)** | -0.003 (0.610) |
| **Feeling depressed** |  |  |  |  |  |  |  |  |
| No | Reference |  | Reference |  | Reference |  | Reference |  |
| Yes | **-0.046 (<0.001)** | **-0.036 (<0.001)** | **-0.014 (0.026)** | -0.004 (0.475) | -0.011 (0.070) | 0.007 (0.265) | 0.008 (0.171) | **0.020 (<0.001)** |
| **Depression** |  |  |  |  |  |  |  |  |
| No | Reference |  | Reference |  | Reference |  | Reference |  |
| 0-4 items | **0.017 (0.006)** | **0.020 (0.001)** | 0.000 (0.944) | -0.002 (0.714) | 0.011 (0.076) | 0.008 (0.173) | **0.022 (<0.001)** | **0.016 (0.005)** |
| 5 items or more | **-0.084 (<0.001)** | **-0.073 (<0.001)** | **-0.020 (0.002)** | -0.004 (0.540) | **-0.027 (<0.001)** | 0.003 (0.658) | -0.008 (0.224) | **0.015 (0.010)** |
| β: Standardized β-Coefficient (one unit is equal to a standard deviation change in the Life’s Essential 8.); LE8: Life’s Essential 8; p: P value.  Model 2: associations were adjusted for sex, age, site, education level and marital status.  Model 3: associations were adjusted for sex, age, site, education level, marital status, alcohol, BMI (except for LE8 BMI), diet, physical activity, smoke, and sleep. | | | | | | | | |
